# Next generation of microbial inoculants for agriculture and bioremediation

**DOI:** 10.1111/1751-7915.12448

**Published:** 2016-10-28

**Authors:** Antonino Baez‐Rogelio, Yolanda Elizabeth Morales‐García, Verónica Quintero‐Hernández, Jesús Muñoz‐Rojas

**Affiliations:** ^1^ Laboratorio de Ecología Molecular MicrobianaCentro de Investigaciones en Ciencias MicrobiológicasInstituto de CienciasBenemérita Universidad Autónoma de Puebla (BUAP)PueblaMexico; ^2^ BiotecnologíaEscuela de Biología‐BUAPPueblaMexico; ^3^ CONACYT‐BUAPPueblaMexico

## Abstract

In this Crystal Ball we describe the negative effects of the scheme of intensive agriculture of the green revolution technology. To recover the contaminated soils derived from intensive farming is necessary introduce new successful technologies to replace the use of chemical fertilizer and toxic pesticides by organic fertilizers and biological control agents. Our principal speculation is that in a short time authors in the field of PGPB and bioremediation will be expanding the knowledge on the development of different formulations containing super‐bacteria or a mixture of super‐bacteria able to provide beneficial effect for agriculture and bioremediation.

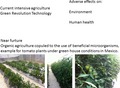

The global population has grown dramatically increasing the needs for food (Tilman *et al*., 2002). To satisfy the food needs, farmers of all countries have implemented the green revolution technology. However, green revolution have provoked several adverse effects to the environment due to indiscriminate use of pesticides, herbicides and nitrogen fertilizers; the use of improved varieties and transgenic, among others (Tilman, 1998). Many types of herbicides and pesticides have carcinogenicity potential (Zahn and Ward, 1998; Damalas and Eleftherohorinos, 2011). Even though some of those toxic compounds have been prohibited in European countries, several of them are still being applied to the crops in different regions of the world. In addition, application of those products can promote the accumulation of toxic compounds in soils. Although little is known about this topic, we can infer that the crop plants are able to absorb these compounds from soil, representing a latent problem to the human health and environment. In fact, it has been demonstrated that some pesticides can be absorbed from soil by potatoes (Juraske *et al*., 2011) and the highly recalcitrant compound TNT can be absorbed by maize plants (Van Dillewijn *et al*., 2007). Nonetheless, something even more important, it will be determined if TNT is able to travel to maize fruits or just stay in the steam of the plants. In other hand, the indiscriminate use of chemical fertilizers has negatively affected to the environment. Only 30% of nitrogen compounds added to the crops can be absorbed by plants, the remaining nitrogen is leached to the groundwater generating eutrophication, NOx gaseous compounds and the concomitant acid rain and detrimental effects on ozone layer (Moiser, 2001). The implementation of improved varieties and transgenic plants to crops increase the productivity. However, this practice could have a negatively impact on the future of the planet, as genetic diversity harboured by those native plants is being displaced (Evenson and Gollin, 2003). As a consequence of damage caused by green revolution, farmers of different places of the world are implementing organic farming schemes based on the past experiences of agriculture and also introducing new technologies to replace the use of chemical fertilizer and toxic pesticides by organic fertilizers and biological control agents. However, the majority of crop fields of the world are still operating under the scheme of intensive agriculture of the green revolution technology and the organic agriculture does not impact much on either economic or environmental aspects (Tilman *et al*., 2002; De Ponti *et al*., 2012; Seufert *et al*., 2012). Therefore, a major effort should be made to increase the positive impact of organic farming practices on both aspects. One possibility could be the synergic effect of combine the organic technology with the use of microorganisms able to carry out both the plant growth promotion and the bioremediation of contaminated soils derived from intensive farming. This synergy maybe of greater impact on the development of sustainable agriculture, farmers could get high yields and additionally they could restore their damaged and contaminated soils.

Beneficial bacteria have been isolated and studied since early nineteenth century and today still exist research groups working in isolation and description of new bacterial species with potential for agriculture, bioremediation, biomedicine and food industry. Plant growth promoting bacteria (PGPB) and bioremediation bacteria have been extensively studied and several molecular mechanisms have been described (Abraham *et al*., 2002; Ramos *et al*., 2005; Lugtemberg and Kamilova, 2009; Bhattacharyya and Jha, 2012). Research studies on molecular dialogue between bacteria and plants has been carried out by some authors showing the wonderful interchange of signals that occur between interacting organisms (Badri *et al*., 2009; Segura and Ramos, 2013). The ability of bacteria to bioremediate toxic compounds and with potential as PGPB suggests that these bacteria could interact effectively with plants in agricultural contaminated soils, carrying out the degradation of pollutants and increasing crop yields.

Despite the large number of reports showing the advantages of the use of PGPB in crops, the application of these microorganisms on the fields is still little explored in comparison to the total amount of agricultural land of the world. Bacterial formulations with PGPB have not always the desired effectiveness (Dobbelaere *et al*., 2001). The capability of microorganisms to promote the growth of plants in crop fields dependent of several factors that limit their effectiveness, for example, soil types, climatic conditions, variety of the crop, bacterial genotype, effectiveness of the bacterial isolate, the proper inoculation technology and others (Bashan, 1998; Bashan *et al*., 2014). When bacteria are in co‐interaction with crop plants, the expression of genes involved in bioremediation and plant growth promotion may be fundamental to obtain the beneficial effect (Segura and Ramos, 2013). In our opinion, these interesting genes could be turned on or off depending on environmental conditions, which suggest that different conditions can occur in the crop fields that may be affecting the gene expression. This could explain why some bacteria improve the growth of plants under laboratory conditions but frequently fails under field conditions or in other cases we observe variable results (Okon and Labandera‐Gonzales, 1994).

In order to ensure the promoting effect of bacteria on plant growth, better adapted bacteria to the conditions where they will be applied have been studied, because they could be more active under that conditions. Authors are focusing in bacteria bearing diverse beneficial abilities, for example bacteria containing genes involved in metabolic pathways for plant growth stimulation and bioremediation. In this regard, *Burkholderia unamae* represent an example of adapted bacteria with potential as PGPB to be applied in Mexican fields. *B. unamae* has the potential to fix nitrogen, produce phytohormones, siderophores and other inhibitory substances, ACC‐deaminase activity and degradation of toxic aromatic compounds (Caballero‐Mellado *et al*., 2004, 2007; Onofre‐Lemus *et al*., 2009). Nevertheless, as we mention before, effective bacteria for the crop field will be those able to express the genes responsible of the plant growth promotion or bioremediation in association with plants under real environmental conditions. However, the key genomic aspects are not completely understood and they should be explored. For example, studies of the kind of promoters and transcriptional factors involved in the regulation of the expressed genes in association with plants are needed. Otherwise, super‐bacteria with the potential to stimulate plant growth under laboratory conditions could fail to provide their beneficial effects to plants under field conditions.

In other hand, bacteria are not alone in nature, therefore a consortium of bacteria could be more effective for plant growth promotion than a single bacterium (Martinez de Oliveira *et al*., 2006; Sundaramoorthy *et al*., 2012). However, the design and formulation of bacterial consortia is not a trivial task, members of the bacterial mixture should be able to coexist without any antagonism among them. In addition, bacteria chosen for the formulation of the bacterial mixtures should bear different abilities to increase the growth of plants, carry out bioremediation, besides to be tolerant to adverse conditions prevalent in the crop fields. Other important feature of why bacterial mixtures could work better than single‐bacterium formulations is because there are higher probability than one member of the bacterial mixture carry out the functional gene expression required for the plant growth promotion.

Putting both strategies together, we could obtain special bacterial mixtures with high probability to obtain a positive effect for bioremediation and high crop yields. In this sense, our principal speculation is that in a short time several authors in the field of PGPB and bioremediation will be expanding the knowledge on the development of different formulations containing super‐bacteria able to provide beneficial effect in association with plants. In addition, a major development of works related to the design of bacterial inoculants in mixture, for agriculture and bioremediation, will be observed. In both cases, it will be desirable to demonstrate that the expression of genes involved in plant growth promotion and bioremediation are working in association with plants under different environmental conditions. For bacterial mixtures, it will be also necessary to demonstrate that they work better than mono‐species formulations.

If the crop yields are constantly increased after every inoculation of beneficial microorganisms, it will be easier to convince farmers around the world to substitute the green revolution technology by the use of microbial inoculants that are friendlier to human health and environment.

## Conflict of Interest

None declared.
